# Effect of *Agrimonia eupatoria* L. and *Origanum vulgare* L. Leaf, Flower, Stem, and Root Extracts on the Survival of *Pseudomonas aeruginosa*

**DOI:** 10.3390/molecules28031019

**Published:** 2023-01-19

**Authors:** Kateřina Bělonožníková, Eliška Sladkovská, Daniel Kavan, Veronika Hýsková, Petr Hodek, Daniel Šmíd, Helena Ryšlavá

**Affiliations:** Department of Biochemistry, Faculty of Science, Charles University, Hlavova 2030, 128 43 Prague, Czech Republic

**Keywords:** *Agrimonia eupatoria* L., *Origanum vulgare* L., *Pseudomonas aeruginosa*, antimicrobial activity, antioxidant capacity, bioluminescence, traditional medicine, chronic pulmonary disease, cystic fibrosis

## Abstract

*Pseudomonas aeruginosa* is one of the most antibiotic multi-resistant bacteria, causing chronic pulmonary disease and leading to respiratory failure and even mortality. Thus, there has been an ever-increasing search for novel and preferably natural antimicrobial compounds. *Agrimonia eupatoria* L. and *Origanum vulgare* L. shoots are commonly used as teas or alcoholic tinctures for their human health-promoting and antibacterial properties. Here, we explored the antimicrobial effects of all plant parts, i.e., leaf, flower, stem, and root extracts, prepared in water or in 60% ethanol, against *P. aeruginosa*. The impact of these extracts on bacterial survival was determined using a luminescent strain of *P. aeruginosa*, which emits light when alive. In addition, the antimicrobial effects were compared with the antioxidant properties and content of phenolic compounds of plant extracts. Ethanolic extracts of *O. vulgare* roots and flowers showed the highest antimicrobial activity, followed by *A. eupatoria* roots. In particular, chlorogenic acid, the ethanolic extract of *O. vulgare* roots contained high levels of protocatechuic acid, hesperidin, shikimic acid, rutin, quercetin, and morin. The synergistic effects of these phenolic compounds and flavonoids may play a key role in the antibacterial activity of teas and tinctures.

## 1. Introduction

Plants have been used since ancient times to treat diseases and improve the health of humans and animals. Plants contain a wide variety of special substances known as secondary metabolites. Some of these substances are formed in the shikimate and phenylpropanoid pathways, such as phenolic acids, flavonoids, lignans, lignins, tannins, terpenoid substances and alkaloids [1,2,3]. In particular, plants known as medicinal plants have a high content of these substances, which are not directly involved in primary metabolism but provide plants with benefits such as UV protection, antibacterial, antifungal or antiviral properties or pollinator attraction. They mainly have antioxidant properties, that is, they have the ability to reduce metabolites in the event of an oxidoreductive imbalance that accompanies oxidative stress. Moreover, these substances quench radicals, including some reactive oxygen species. Along with other properties of plant secondary metabolites, their antioxidant potential is beneficial to animals and humans [1,2,3,4].

Among common medicinal plants, a significantly high antioxidant capacity was detected in *Agrimonia eupatoria* L. and *Origanum vulgare* L. (commonly called oregano) [4]. Interestingly, these two plants have a strong traditional background. As a case in point, the name of *A. eupatoria*, is derived from the Greek word agremone for eye-healing plants [5]. Similarly, *A. eupatoria* extracts were used as the main ingredient of a battlefield cure for bullet wounds in the 15th century, [6] and *O. vulgare* has been used since the time of Hippocrates (5th century B.C.) [7] in the form of teas and tinctures to treat human diseases, more specifically respiratory and digestive problems, headaches and depression, and in the form of ointments for wound healing [8,9]. 

*O. vulgare* is widespread in the Mediterranean region but is found throughout Europe and in much of Asia, including at higher altitudes in the mountains [8]. For its pleasant aroma, *O. vulgare* is used as a spice in culinary and perfumery [9]. Recently, several studies have shown that its composition of active substances includes a high number of volatile and non-volatile compounds and varies as a function of many factors such as geographical location, soil composition, surrounding environment, harvesting season and, above all, plant processing, i.e., extraction method. Essential oils obtained by hydrodistillation contain monocyclic monoterpenes, derivatives of *p*-cymene, such as carvacrol, thymol, acyclic monoterpenes such as linalool, geraniol, myrcene, bicyclic sabinene, pinene, but also sesquiterpenes [7,8,9,10,11]. Non-volatile phenolic compounds, including phenolic acids, especially rosmarinic acid, flavonoids, both free and in the form of glycosides, such as luteolin and apigenin and tannins, are major constituents of water and ethanol extracts [8]. Differences in active compound content may exist in the same medicinal plant from different areas, growing seasons, solar radiation, growth conditions and different plant parts from the same herb [12,13]. For instance, *O. vulgare* collected from different environments showed significant differences in essential oil composition; in plants from the Mediterranean the content of carvacrol and thymol predominates [7,14]. In turn, plants from central and northern Europe usually contain more non-volatile compounds [8].

The essential oils and other extracts of oregano have antimicrobial effects, i.e., the ability to inhibit or stop bacterial growth, as shown in several species of Gram-positive (e.g., *Staphylococcus aureus*, *Bacillus subtilis*, *Enterococcus faecalis*, and *Streptococcus pneumoniae*) and -negative (e.g., *Pseudomonas aeruginosa*, *Escherichia coli*, *Klebsiella pneumoniae*, and *Helicobacter pylori*) bacteria [15]. These antibacterial effects are highly promising, especially considering the ever-increasing number of antibiotic-resistant bacterial strains. Most pathogenic bacteria such as *S. aureus* affect host tissues; they produce proinflammatory cytokines, which trigger inflammation. *O. vulgare* essential oils reduce TNF-alpha, IL-1beta, IL-8 and inhibit NADPH oxidase and lipoxygenase, thereby suppressing inflammatory reactions. These reactions have a positive effect on the skin surface, epidermis and dermis, susceptible to infections [7]. *O. vulgare* essential oil also counteracts biofilm formed by skin bacteria, *Propionibacterium acnes* and *S. aureus*, and effectively protects the skin from acne manifestations [7]. Another positive property of oregano essential oil is the inhibition of collagenase, elastase and hyaluronidase and thus its anti-ageing effect [7]. In addition to its antibacterial properties, oregano essential oil also has fungicidal properties against *Candida albicans*, *Aspergillus flavus* and *Penicillium funiculosum* [9]. The ability of phytochemicals present in *O. vulgare* essential oil to inhibit acetylcholinesterase is correlated with possible treatment of Alzheimer’s disease, also the inhibition of ɑ-amylase and ɑ-glucosidase enzymes is beneficial for adjusting blood glucose level [9].

The second plant of our interest, *A. eupatoria*, a perennial herb from the Rosaceae family, is one of the most common medicinal plants in Central Europe, which is also found across Asia Minor and North Africa [6]. *A. eupatoria* tinctures, teas, gargles or wash are used in traditional medicine for their anti-inflammatory, antibacterial, antiseptic and antipyretic properties to cure pulmonary diseases, bronchitis and sore throats [6,16]. *A. eupatoria* positive effects are, however, associated with the alleviating of *diabetes mellitus*, urinary infections, gastrointestinal diseases, obesity, viral diseases and even cancer. At the same time, no severe adverse effects have been reported after consumption of aqueous extracts of *A. eupatoria*, which is considered safe and generally well tolerated [16,17]. Furthermore, *A. eupatoria* incorporated into a two-layer cotton material coated with nanofibers for wound dressing showed no cytotoxicity against the normal human dermal fibroblast cells [18].

*A. eupatoria* effects correlate with its high content of antioxidants, especially phenylpropanoids (tannins, flavonoids, phenolic acids, and anthocyanidins) and terpenoids (some of which are essential volatile oils). These components, such as thymol, α- and β-pinene, α- and β-cedrene, D-limonene, eucalyptol, bergamot oil, geraniol acetate, linalool and hexanal, are responsible for its characteristic odor and taste. Ghobadi Pour et al. (2021) excellently summarized 68 terpenoids and 73 phenolic and flavonoid compounds in *A. eupatoria* [6]. Among phenolic and flavonoid compounds, agrimonin, derivatives of apigenin, quinic acid, kaempferol, luteolin, procyanidin, quercetin, quercitrin, and rutin were found [6]. The antibacterial activity of water, alcoholic or acetone *A. eupatoria* extracts against Gram-positive (such as *Staphylococcus aureus*) and Gram-negative bacteria (e.g., *Vibrio cholerae*, *Pseudomonas aeruginosa*, and *Escherichia coli*) was determined [19,20,21].

Currently, our research is focused on *Pseudomonas aeruginosa*, an opportunistic pathogen that frequently occurs in the environment. Since *P. aeruginosa* is one of the most antibiotic resistant bacteria, considerable research efforts have been made to develop antimicrobial agents. Patients suffering from cystic fibrosis are highly susceptible to *P. aeruginosa*. The lungs of approximately three-quarters of adult patients with cystic fibrosis are infected with this microorganism. Thus, *P. aeruginosa* is a major cause of chronic pulmonary infection, leading to respiratory failure and mortality in cystic fibrosis patients [22]. Moreover, due to long-term and continued antibiotic administration, cystic fibrosis patients are at increased risk of developing *P. aeruginosa* infections with multidrug-resistant bacteria [23], in addition to the side effects of the antibiotics.

In view of the increasing antibiotic resistance of *P. aeruginosa* strains burdening patients with cystic fibrosis, we must look into plant extracts for their antibacterial effect towards alleviating *P. aeruginosa* infections. The advantage of herbal-based traditional medicine lies in its weaker side effects than pharmaceutical drugs. The effects of *O. vulgare* and *A. eupatoria* on *P. aeruginosa* bacteria have already been investigated, albeit focusing on essential oils prepared especially from shoots [7,9,10]. Thus, the present study aims at a complex examination of the anti-*P. aeruginosa* potency of water (teas) and 60% ethanolic extracts (tinctures) from all parts of this plant, i.e., leaves, flowers, stems and roots. For this purpose, we characterized its ability to stop *P. aeruginosa* growth and the antioxidant properties of its extracts.

## 2. Results

### 2.1. Total Phenolics, Flavonoids, and Antioxidant Capacity of A. eupatoria and O. vulgare

**Figure 1:**
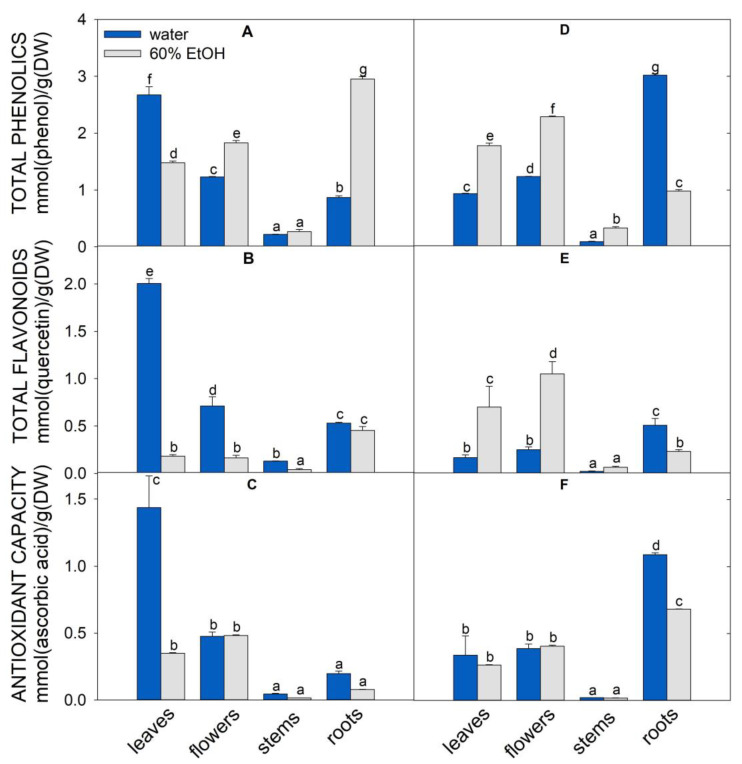
Characterization of *A. eupatoria* (**A**–**C**) and *O. vulgare* (**D**–**F**) extracts prepared in water (blue columns) or 60% ethanol (gray columns). Total phenolics (**A**,**D**), total flavonoids (**B**,**E**), antioxidant capacity determined by FRAP assay (**C**,**F**). The quantity is expressed as mmol per gram of dry plant matter. The values show the mean ± SD. Letters above each bar denote significant differences (*p* ≤ 0.05) among the groups according to ANOVA (Holm–Sidak method). Abbreviations: DW, dry weight (lyophilizate); FRAP, ferric ion reducing power.

Traditionally, the shoots of *A. eupatoria* and *O. vulgare* are used and known for their beneficial effects on human health. In our complex study, we also included less studied flowers, stems, and roots. Individual fresh plant parts were lyophilized, and the dry weight was determined and expressed as percentage (Figure S1). In all plant parts, the dry weight of *O. vulgare* was higher than that of *A. eupatoria.* The flowers showed a slightly higher dry weight than the leaves. The leaves and roots showed similar dry weight in both plants (Figure S1). Two sets of samples, water and 60% ethanolic extracts, were prepared from lyophilized leaves, flowers, stems, and roots of both plants to determine the total content of phenolic compounds and flavonoids and the antioxidant capacity (Figure 1). While water extraction of total phenolic compounds and flavonoids from *A. eupatoria* leaves was more effective, more phenylpropanoids were extracted with 60% ethanol from *O. vulgare* leaves. Moreover, *O. vulgare* roots had a higher content of phenolics and flavonoids and a higher antioxidant capacity than *A. eupatoria* leaves. Conversely, the stems of both plants were unsuitable sources of secondary metabolites because they showed the lowest content of these compounds and the lowest antioxidant capacity. The water extracts of *A. eupatoria* flowers contained approximately 2.2 and 2.8 times less total phenolic compounds and flavonoids than those of leaves, respectively. In flowers, the content of these compounds was slightly higher in *O. vulgare* ethanolic extracts (Figure 1). In summary, the highest antioxidant capacity was found in water extracts of *A. eupatoria* leaves and *O. vulgare* roots (Figure 1).

### 2.2. Antimicrobial Activity of A. eupatoria and O. vulgare Water and Ethanolic Extracts against P. aeruginosa

**Figure 2:**
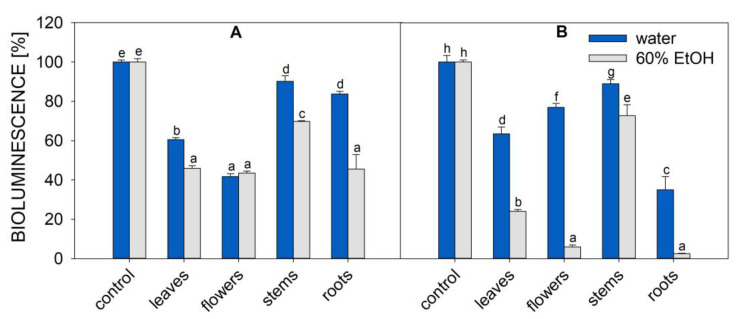
Bioluminescence of PA-Lux (indicating bacteria viability) in the presence of *A. eupatoria* (**A**) and *O. vulgare* (**B**) extracts prepared in water (blue columns) or 60% ethanol (grey columns). Evaporated extracts dissolved in LB medium were incubated with PA-Lux for 1 h at 37 °C. The plant extract in LB medium (100 μL) was diluted with an equal volume of PA-Lux suspension. Negative control (100%) represents the bioluminescence of PA-Lux with LB medium only. The values show the mean ± SD. Different letters next to each bar denote significant differences (*p* ≤ 0.05) among the plant groups according to ANOVA (Holm–Sidak method). Abbreviations: LB, Luria broth; PA-Lux, luminescent strain of *P. aeruginosa*.

The anti-*P. aeruginosa* efficacy of plant extracts was assessed using the *P. aeruginosa* strain with a Tn5-luxCDABE transposon inserted into the fliC gene (PA-Lux) [24]. It is a convenient tool since PA-Lux, when growing, constitutively expresses luminescence [25]. Thus, the antimicrobial potency of the extracts is directly reflected in the decrease in bacteria luminescence. Figure 2 shows the luminescence of PA-Lux, which corresponds to the average amount of live bacteria in the presence of aqueous and ethanolic extracts of *O. vulgare* and *A. eupatoria* prepared from individual plant parts.

In the negative control (100% of bioluminescence), the plant extract was replaced with Luria broth (LB) medium. All extracts of *O. vulgare* and *A. eupatoria* plant parts decreased bioluminescence to some extent, demonstrating their inhibitory effect on PA-Lux growth.

Among all extracts tested in this study, the samples prepared by ethanolic extraction of flowers and roots of *Origanum vulgare* showed the best overall inhibition of PA-Lux growth (residual luminescence was 6 and 3%, respectively, Figure 2). In *A. eupatoria* extract, except for stems, all plant parts showed similar PA-Lux growth inhibition rates. After the incubation of various *A. eupatoria* ethanolic extracts with PA-Lux, the average luminescence decreased to 46–44% of control for root, leaf, and flower extracts, but the lowest growth inhibition was obtained when using stem extracts from both plants, regardless of extraction method. For *A. eupatoria*, the average luminescence of stems was 90% for aqueous extracts and 70% for ethanolic extracts. For *O. vulgare*, the average luminescence of stems was 89% for aqueous extracts and 73% for ethanolic extracts. In all cases, except for *A. eupatoria* flowers, water extracts were less effective in suppressing PA-Lux growth than ethanolic extracts. However, water extracts of *O. vulgare* roots and leaves and *A. eupatoria* leaves and flowers also significantly reduced PA-Lux growth (Figure 2).

### 2.3. Determination of MIC50 Values for A. eupatoria and O. vulgare Water and Ethanolic Extracts

**Figure 3:**
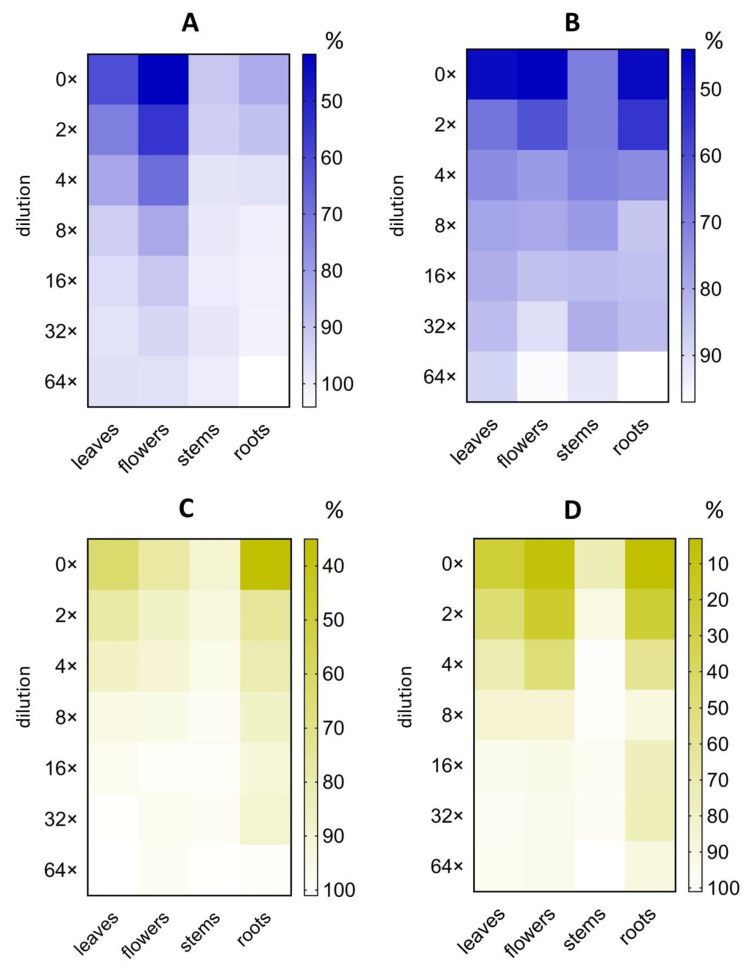
The effect of serial dilutions of plant extracts on PA-Lux bioluminescence. *A. eupatoria* (blue) as water extract (**A**) and 60% ethanol extract (**B**) and *O. vulgare* (yellow) as water extract (**C**) and 60% ethanol extract (**D**). Evaporated extracts dissolved in LB medium were incubated with PA-Lux for 1 h at 37 °C. The plant extract in LB medium (100 μL) was diluted with an equal volume of PA-Lux suspension. Negative control (100%) represents the bioluminescence of PA-Lux with LB medium only. The values show the mean ± SD. Abbreviations: LB, Luria broth; PA-Lux, luminescent strain of *P. aeruginosa*.

The minimal inhibitory concentration that caused a 50% decrease in PA-Lux bioluminescence (MIC50), that is, in *P. aeruginosa* viability, was calculated based on the variation of PA-Lux bioluminescence as a function of plant extract dilution (Figure 3, Table 1).

MIC50 was expressed in two ways, i.e., based on either the amount of lyophilized plant material, or evaporated extract per ml in the incubation mixture with PA-Lux. The lowest MIC50 (lyophilizate) values were determined for *O. vulgare* ethanolic extracts of roots (4 mg/mL) and flowers (7 mg/mL), followed by water root extract (8 mg/mL). For *A. eupatoria* the highest value was 10 mg/mL for water extract of flowers. The lowest MIC50 (evaporate) values were found among ethanolic extracts from roots and flowers of *O. vulgare* plants (2.3 mg/mL and 4.5 mg/mL) and from roots and flowers of *A. eupatoria* plants (17.3 mg/mL and 19.7 mg/mL). Concerning water extracts, the lowest MIC50 (evaporate) showed extracts of stems of *A. eupatoria* and *O. vulgare* (10.1 and 14.3 mg/mL) and the extract of *O. vulgare* stem and roots (7.1 and 12.4 mg/mL) (Figure 3, Table 1). For comparison, sodium azide, a prototypic antibacterial preservative compound was used. MIC50 of sodium azide as positive control reached 0.05 mg/mL (0.9 ± 0.3 μmol/mL) (Figure S2). Unexpectedly, the roots prepared via the ethanolic extraction of both plants excelled at inhibiting the PA-Lux growth (low MIC50), especially those of *O. vulgare* roots.

### 2.4. Correlation Analysis between Plant Characteristics and P. aeruginosa Viability

In the correlation analysis (Pearson’s coefficients, Supplementary Materials Figure S3), we compared the value of bioluminescence, MIC50 (lyophilizate) and the content of flavonoids and phenolics and the antioxidant capacity of both plants for the same extraction method. For water extracts, the positive correlation coefficients were high between phenolics and antioxidant capacity (0.95), flavonoids and antioxidant capacity (0.83). In addition, we found a high negative correlation between MIC50 (lyophilizate) and phenolics (−0.98), MIC50 (lyophilizate) and antioxidant capacity (−0.93, Figure S3A). For ethanolic extraction, the correlation coefficients were significantly lower (Figure S3B). Overall, ethanolic extracts contain a wider variety of compounds that inhibit PA-Lux growth, with high efficiency even at low concentrations, but contribute less to the antioxidant capacity or do not count as phenolics. Predominantly, the root extracts of both plants stand out for their properties.

### 2.5. Identification of Phenolic Compounds in A. eupatoria and O. vulgare Water and Ethanolic Extracts

The LC-MS comparative analysis of phenolic compounds in all extracts was carried out with 23 standards. Data outlined in Table 2 highlight that *A. eupatoria* contain higher concentrations of protocatechuic acid, quinic acid, phenylalanine, shikimic acid, and dihydromyricetin in leaves than in other plant parts, as shown in both water and ethanolic extracts. Conversely, *A. eupatoria* roots were the richest source of caffeic acid, vanillin, gallic acid, sinapic acid, morin, and *trans*-cinnamic acid of this plant. Flowers and stems of *A. eupatoria* also contained naringin.

*O. vulgare* leaf and flower extracts contained high amounts of quercitrin, quinic acid, chrysin, and protocatechuic acid (Table 2), whereas *O. vulgare* roots contained high amounts of chlorogenic acid, protocatechuic acid, hesperidin, shikimic acid, rutin, quercetin, and morin.

**Figure 4:**
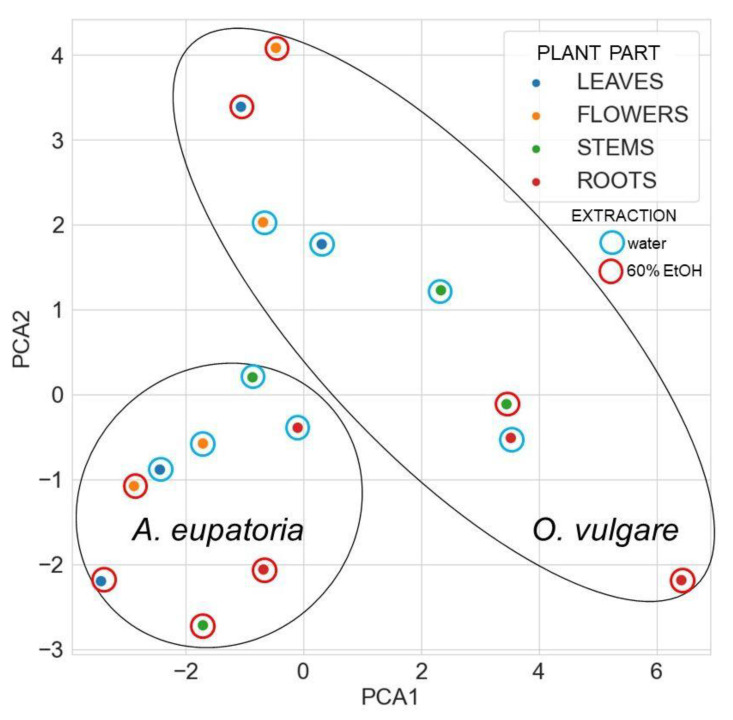
The principal component analysis of identified secondary metabolites in water or 60% ethanol extracts of *A. eupatoria* and *O. vulgare.* The analysis compares the composition in the tested extracts against PA-Lux. Data were plotted using the Seaborn library for making statistical graphics in Python [26]. Abbreviations: PA-Lux, luminescent strain of *P. aeruginosa*.

Principal component analysis based on detected secondary metabolites revealed that all *A. eupatoria* extracts differ from *O*. *vulgare* extracts (Figure 4).

**Figure 5:**
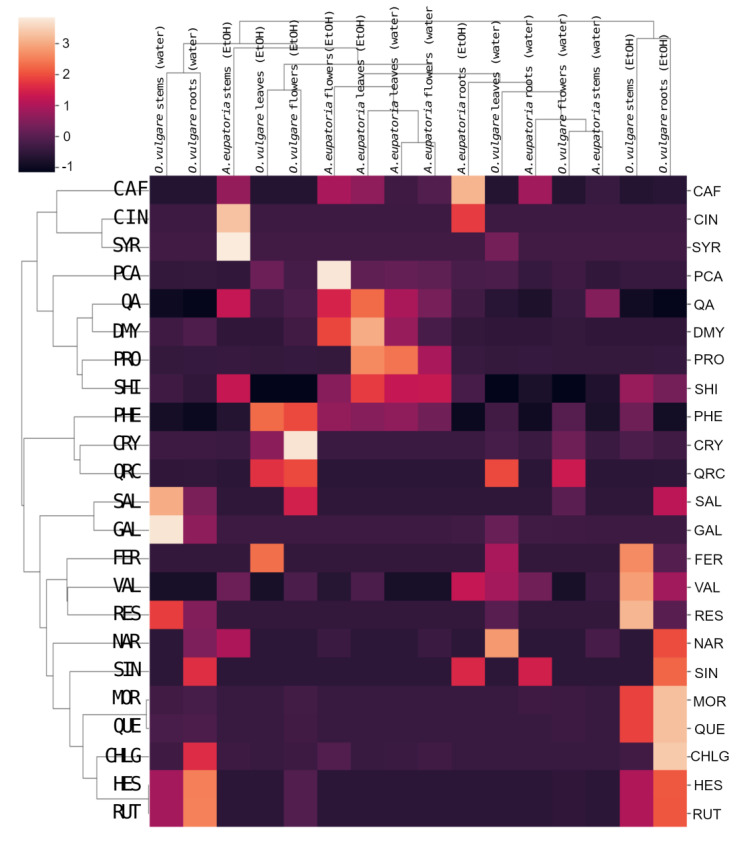
Heatmap with dendrogram of hierarchical clustering of secondary metabolites identified in water or 60% ethanol extracts of *A. eupatoria* and *O. vulgare*. The analysis compares the composition of the extracts against PA-Lux. Data were plotted using the Seaborn library for statistical graphics in Python [26]. Abbreviations: CAF, caffeic acid; CHLG, chlorogenic acid; CIN, *trans*-cinnamic acid; CRY, chrysin; DMY, dihydromyricetin; FER, *trans*-ferulic acid; GAL, gallic acid; HES, hesperidin; MOR, morin; NAR, naringin; PA-Lux, luminescent strain of *P. aeruginosa*; PCA, *p*-coumaric acid; PHE, phenylalanine; PRO, protocatechuic acid; QA, quinic acid; QRC, quercitrin; QUE, quercetin; RES, resveratrol; RUT, rutin; SAL, salicylic acid; SHI, shikimic acid; SIN, sinapic acid; SYR, syringic acid; VAL, vanillin.

For *A. eupatoria*, water and ethanol extracts are found in different areas of its cluster in Figure 4, but its leaf water extract and flower ethanol extract lie close to each other, indicating some similarity. In turn, the *O. vulgare* root ethanol extract significantly differs from the other extracts. Nevertheless, some similarity is found between the *O. vulgare* leaf and flower ethanol extracts and between the stem ethanolic extract and the water root extract. In the heatmap with a dendrogram of hierarchical clustering of secondary metabolites, the compounds form three main clusters, whereas plant extracts form two main clusters (Figure 5).

The root extract of *O. vulgare* has a unique content of naringin, sinapic acid, morin, quercetin, chlorogenic acid, hesperidin, and rutin, whereas the flower extract of *O. vulgare* differs from others primarily in its phenylalanine, chrysin, quercitrin, and salicylic acid content (Figure 5). Both water and ethanolic extracts of *A. eupatoria* leaves and flowers cluster closely and stand out for their *p*-coumaric acid, quinic acid, dihydromyricetin, protocatechuic acid, shikimic acid, and phenylalanine content (Figure 5).

## 3. Discussion

Due to the increasing resistance of a high number of pathogens to antibiotics, the use of natural compounds has been gathering interest among researchers. *P. aeruginosa* and especially its strains resistant to antibiotics cause serious respiratory problems to immunocompromised individuals and patients with cystic fibrosis [22,23]. Thanks to their antioxidant, antibacterial, and anti-inflammatory properties, medicinal plants, in the form of teas (infusions), inhalation solution or gargle, may prevent infection or reduce bacterial load in the respiratory tract of chronically ill patients. Accordingly, scientists continuously seek to develop novel methods for testing antimicrobial activity.

Standard antimicrobial bioassays such as disk-diffusion, well diffusion and broth or agar dilution are well known and widely used, but they require viable counting and are lengthy and labor intensive [27,28]. In the present study, we used a bioluminescent strain of *P. aeruginosa* (PA-Lux), which emits light when alive because bioluminescence is a non-disruptive and elegant method for high-throughput screening of PA-Lux growth. Firstly, we validated our experimental set-up with sodium azide as a typical antibacterial preservative compound (Figure S2). The incubation time was optimized based on the effect of sodium azide on PA-Lux, which was used in a wide range of concentrations, from non-lethal to lethal. The luminescence of PA-Lux determined 60 min after the assay initiation was well suited for assessing the antibacterial effect. As the effectiveness of inhibition also depends on the bacteria’s living conditions, in our experiments, all perturbants (sodium azide and plant evaporates) were diluted with LB medium to allow the PA-Lux growth, not limited in nutrients within the incubation period.

Based on our previous study aimed at characterizing potential medicinal plants from Central Europe [4], we chose *A. eupatoria* and *O. vulgare* as a source of natural *P. aeruginosa* inhibitors because they showed the highest amounts of antioxidants in leaves. In addition, our present data indicated significant amounts of total phenolics and flavonoids and antioxidant capacity in roots and flowers (Figure 1). Generally, *A. eupatoria* and *O. vulgare* are traditionally used in medicine, and oregano is commonly used as flavoring herb and food additive.

Most studies focus on essential oils highly concentrated in complex mixtures, mainly composed of terpenes and other compounds, e.g., aldehydes, phenols, esters, and alcohols, among other compounds [29]. *O. vulgare* essential oils, in particular, have been tested for their antibacterial activity [9,14,15,30,31]. For a more comprehensive analysis of potential antibacterial plant constituents, and not merely those present in essential oils, we tested *A. eupatoria* and *O. vulgare* water extracts, simulating teas, and ethanolic extracts, representing tinctures. Leaching dried plant material (lyophilizate) in boiling water or ethanol is an easy and affordable way of preparing medicinal solutions at home and thus readily available and very popular. Moreover, in addition to commonly used aerial plant parts (mainly leaves), we analyzed the secondary metabolite composition, antimicrobial effects, and antioxidant capacity of different plant parts (leaves, flowers, stems and roots).

MIC50 is frequently used to express the effectiveness of microbial growth inhibition. However, comparisons between laboratories are often limited because their quantification of the inhibitor sample is not uniform. MIC50 values are also affected by drying and extraction conditions, including solvent, time, and temperature, which are not standardized. Table 1 outlines our MIC50 values, which were calculated based on the amount of plant lyophilizate or evaporate in the incubation mixture with PA-Lux. Surprisingly, the highest antimicrobial effect, and thus the lowest MIC50 (lyophilizate), was found in ethanolic extracts of *O. vulgare* roots (4 mg/mL) and flowers (7 mg/mL) (see Figure 2 and Figure 3; Table 1). To our knowledge, this is the first study to report the MIC50 (lyophilizate) of *A. eupatoria* root extracts (20 and 55 mg/mL for water and ethanolic extract, respectively) against *P. aeruginosa*.

Muruzovic et al. (2016) assessed the effects of acetone, ethyl acetate, ethanolic and water extracts of *A. eupatoria* shoots on the growth inhibition of Gram-positive and Gram-negative bacteria for 24 h using resazurin as viability detection [19]. Against *P. aeruginosa,* they found MIC (defined as no bacterial growth) values for aqueous and ethanol extracts of 10 and 1.25 mg/mL, respectively. For essential oils, the reported MIC values are usually lower, most likely due to the highly concentrated volatile and hydrophobic compounds. For example, *O. vulgare* essential oil obtained by hydrodistillation of leaves showed quite low MIC (no bacterial growth) against *P. aeruginosa*, 0.15–0.06 mg/mL, matching the effect of antibiotics [31,32,33].

The effective compounds behind this antimicrobial effect were identified by LC-MS of water and ethanol extracts of *A. eupatoria* and *O. vulgare*. Although our analysis was limited by the number of standards of secondary metabolites (23), we were able to identify extract constituents and associations with antimicrobial effects (Table 2, Figure 5 and Figure S4). These compounds mainly included phenolic acids (shikimic acid, *trans*-cinnamic acid, caffeic acid, *trans*-ferulic acid, gallic acid, *p*-coumaric acid, protocatechuic acid, sinapic and syringic acid, vanillin), flavonoids (quercetin, chrysin, dihydromyricetin, and morin), and flavonoid glycosides (rutin, naringin, hesperidin, and quercitrin). Quercitrin, quinic acid, chrysin, and protocatechuic acid were detected in *O. vulgare* leaves and flowers, whereas chlorogenic acid, protocatechuic acid, hesperidin, shikimic acid, rutin, quercetin, and morin were detected in roots (Table 2, Figure 5). The ethanolic extract of *O. vulgare* roots was the most effective, decreasing the luminescence of PA-Lux to only 3% (Figure 2).

From these results, two key questions emerge as to which compounds are involved and how is the antimicrobial effect triggered. In literature, the mechanism of antibacterial action of *O. vulgare* has been mainly investigated in essential oils rich in hydrophobic volatile compounds, which can penetrate through the cell wall and membrane into the bacterial cell inhibit bacterial enzymes and thus affect bacterial metabolism [7,8,9,10,11]. In addition, essential oils can inhibit efflux pumps, which is especially important for resistant strains of bacteria when essential oils can be applied in combination with antibiotics. Predominantly, thymol and carvacrol (monoterpenes) are responsible for essential oil antimicrobial effect, they can damage the bacterial membrane, reduce pH gradient and hence decrease the ATP concentration [8]. Essential oils, through their components, damage bacterial cell walls and membranes. As a result, key molecules such as proteins, nucleic acids or ATP exit the bacterium, and in some cases, reactive oxygen species are also formed, causing oxidative stress and lipid peroxidation [15].

Our extracts prepared as teas and tinctures contained predominantly non-volatile compounds, such as phenolics that function at the surface of membranes, depending on lipophilicity and on electronic and charge properties [34,35]. Phenol (in vitro prototype of phenolics) changes membrane functioning, affects protein-to-lipid ratios in the membrane and induces the efflux of potassium ions. Cell wall lysis has also been reported in bacteria exposed to phenolics [35]. In plant extracts, which represent mixtures of various (not only) secondary metabolites, synergistic events, such as adsorption of polyphenols to bacterial membranes with membrane disruption and subsequent leakage of cellular contents combined with the generation of hydroperoxides from polyphenols, most likely determine the resulting antimicrobial activity [34,35].

In our experiments, the ethanolic extract of *O. vulgare* roots (followed by flowers) was the most potent PA-Lux growth inhibitor (Figure 2). This extract contained the highest amount of chlorogenic acid, hesperidin, morin, quercetin, rutin, and sinapic acid, i.e., a mixture of phenolics and flavonoids (Table 2, Figure 5). We compared our results with published data to identify the compounds that could be involved in this antimicrobial activity.

Chlorogenic acid is a conjugate of caffeic and quinic acid, also reported with antibacterial activity. These phenolic acids were found in extracts of both *A. eupatoria* and *O. vulgare*, but the highest amounts were found in the extracts of *O. vulgare* roots, where the highest inhibition of PA-Lux growth was determined (Figure 5, Table 2). Chlorogenic acid is present in many plant families such as Lamiaceae, Asteraceae, Solanaceae, Rosaceae and others, and is responsible for antioxidant, anti-inflammatory, anti-diabetic and antimicrobial effects [36]. Extracts from shoots of some conifers containing chlorogenic and caffeic acids showed significant antimicrobial activity [37]. Pure chlorogenic acid inhibited the growth of both Gram-positive and Gram-negative bacteria, including multi-drug resistant strains of *P. aeruginosa*, whereas *Klebsiella pneumoniae* was the most sensitive [38,39]. Both chlorogenic acid and caffeic acid were also effective against resistant strains of *Stenotrophomonas maltophilia* and mitigated biofilm formation [40]. Sinapic acid was detected only in the root extracts of both plants (Figure 5, Table 2). Sinapic acid showed selective antibacterial activity, inhibiting Gram-positive (*S. aureus*, *Listeria monocytogenes*) and Gram-negative bacteria (*E. coli*, *P. fluorescens*, *Salmonella enterica*), whereas *Lactobacillus plantarum* (G+) was not affected [41,42]. Such selective inhibition could be advantageous for using sinapic acid as food preservative to eliminate foodborne pathogens without affecting the growth of beneficial lactic acid bacteria, which are used as starter cultures, protective cultures, or probiotics [41,42].

Ethanolic root extracts of *O. vulgare* were rich in flavonoid quercetin, which was below the detection limit in all *A. eupatoria* extracts (Table 2, Figure 5). Quercetin is known to inhibit the growth of a wide range of Gram-positive and -negative bacteria (such as *P. aeruginosa*), and its antimicrobial actions include damage to cell membranes, changes in membrane permeability, inhibition of nucleic acid and protein synthesis, reduction of expression of virulence factors, mitochondrial dysfunction, in addition to preventing biofilm formation [43]. Arima et al. (2002) showed that quercetin–quercitrin, quercetin–morin, and quercetin–rutin combinations were much more active against *Salmonella enteritidis* and *Bacillus cereus* than these flavonoids alone. Although rutin showed no antibacterial effect alone, it enhanced the antibacterial activities of quercetin and morin [44]. In the correlation analysis of all secondary metabolites identified in our extract (Figure S4), we found significant positive correlations between quercetin and morin (1) and between rutin and hesperidin (1), which were also predominantly detected in *O. vulgare* root, stem and flower extracts (Table 2). Furthermore, chlorogenic acid was strongly correlated with rutin (0.8), hesperidin (0.8) and quercetin (0.75). All *O. vulgare* extracts also contained chrysin, but the concentration of this flavonoid was under the limit of detection in *A. eupatoria* extracts (Table 2). Xie et al. (2022) found that a phosphate ester derivative of chrysin was effective against *P. aeruginosa* [45]. Therefore, chrysin may also be important for the antibacterial activity of *O. vulgare* extracts.

For *A. eupatoria,* the highest inhibition of PA-Lux was elicited by the ethanolic extracts of leaves, flowers and roots, which differed in their composition of secondary metabolites from *O. vulgare* extracts. However, some of them are also known to exert antimicrobial effects. Extracts of *A. eupatoria* leaves and flowers contained higher amounts of *p*-coumaric acid than extracts of *O. vulgare* (Table 2). *p*-Coumaric acid was tested against a wide range of Gram-positive and -negative bacteria. The results showed that *p*-coumaric acid killed the pathogenic strain (*Shigella dysenteriae*) through a dual mechanism of bactericidal activity. On the one hand, *p*-coumaric acid irreversibly changes cell membrane permeability, causing cells to lose the ability to maintain cytoplasm macromolecules. On the other hand, *p*-coumaric acid binds to DNA, inhibiting cellular functions [46].

Quinic and shikimic acids have been shown to play a key role in the fight against *S. aureus* [47]. These compounds alter membrane composition, fluidity, protein function, and oxidative phosphorylation. Intracellularly, they affect ribosome function and aminoacyl-tRNA synthesis, thereby reducing protein synthesis. In addition, shikimic acid alters the regulation of the pyruvate dehydrogenase complex and the citrate cycle, whereas quinic acid inhibits lysine and peptidoglycan synthesis, which leads to cell wall reduction and primarily affects cell division [47]. Thus, we hypothesize that quinic acid could act against PA-Lux and that shikimic acid may be not only an intermediate in the synthesis of aromatic amino acids but also involved in the antimicrobial effects of *A. eupatoria* extracts.

The lateral pathway of the shikimate pathway forms protocatechuic acid in plants [48]. The water extracts of *A. eupatoria* leaves and flowers contained high concentrations of this acid (Table 2). Protocatechuic acid also showed antibacterial activity against *P. aeruginosa*, *E. coli*, and *S. aureus* by increasing oxidative stress and lipid peroxidation, by reducing glutathione depletion and by inducing DNA fragmentation, leading to cell death [49].

Based on our data, we suppose that the antimicrobial effect depends on the composition of individual secondary metabolites in extracts and their concurring interactions with bacteria. We also measured the total antioxidant capacity, phenolics and flavonoids using standard methods. The results did not entirely match the anti-PA-Lux effects. The water extracts of *A. eupatoria* leaves and *O. vulgare* roots showed the highest antioxidant capacity (Figure 1), in line with the concentration of phenolics and flavonoids (Figure S3). However, a negative correlation (−0.77) was found between PA-Lux survival and the concentrations of phenolics in water extracts. Similarly, the correlation was still weak (−0.45) for ethanolic extracts even though they were more effective in suppressing PA-Lux growth. Because the anti-PA-Lux effect cannot be easily attributed to a particular compound or class of compounds, synergism and concurrent mechanisms of action likely account for these antimicrobial effects.

## 4. Materials and Methods

### 4.1. Plant Extracts

*Agrimonia eupatoria* L. and *Origanum vulgare* L. (cv. *Aureum Variegata*) were grown in the Czech Republic (GPS: 50.1751942N, 15.8584878E) in the garden. Plants (approx. 50 plants each) were harvested in July 2022 during the flowering stage and divided into leaves, flowers, stems and roots. Then, plant parts were lyophilized overnight (Finn-Aqua Lyovac GT2E, Tuusula, Finland). Water extracts were prepared as a simulation of tea, i.e., 0.5 g of dry plant material (lyophilizate) was submerged in 50 mL of boiling water for 15 min. Ethanolic extracts were prepared as tinctures, i.e., 0.5 g of dry plant material (lyophilizate) was submerged in 10 mL of 60%(*v*/*v*) ethanol for 7 days. Subsequently, 1.5 mL of extract was evaporated to dryness using a rotary evaporator (Labconco, Kansas City, MO, USA) at 37 °C. The resulting dry matter (evaporate) was dissolved in 630 μL of Luria broth (LB) with 20 mM glucose and filtered through a 0.22 μm syringe filter (VWR, Radnor, Pennsylvania, USA). To determine the concentrations of total phenolics, flavonoids and antioxidant capacity, the evaporate was dissolved in deionized water.

### 4.2. Total Phenolics and Flavonoids

The content of total phenolic compounds was determined using the standard Folin-Ciocâlteu colorimetric method with slight modification according to [4]. Phenol was used as a calibration standard. The total flavonoids were assayed by a modified Dowd colorimetric method with quercetin as a calibration standard [50]. The calibration curves can be found in Figure S6A,B.

### 4.3. Antioxidant Capacity

The ferric ion reduction antioxidant power assay (FRAP) was performed as in [4]. Ascorbic acid was used as a calibration standard. The calibration curve can be found in Figure S6C.

### 4.4. Analysis of Secondary Metabolites

Secondary metabolites were analyzed by reversed-phase liquid chromatography coupled to electrospray mass spectrometry (LC-MS) [51,52]. The plant sample after evaporation (prepared as above) was dissolved in 10% methanol containing 0.01% formic acid and centrifuged at 12,000 g for 10 min and filtered through PES syringe filter 0.22 µm (VWR, Radnor, PA, USA). The resulting solution was analyzed on a LC system 1290 Infinity II (Agilent, Santa Clara, CA, USA) interfaced to Q-TOF maXis II (Bruker, Billerica, MA, USA). A Zorbax C18 reverse-phase silica-based column was used for separation (150 × 2.1 mm, 3.5 μm; Agilent, Santa Clara, CA, USA). The compound quantities were compared with their respective standard curves. The standards were purchased from Sigma-Aldrich (USA) and Carl Roth (Germany) in ≥98.0% purity (HPLC).

### 4.5. Microorganism Strain and Culture Conditions

The preparation of *P. aeruginosa* (strain H1001) with a Tn5-luxCDABE transposon inserted into the fliC gene (further as PA-Lux) (generous gift from Dr. Hancock, Centre for Microbial Diseases and Immunity Research, University of British Columbia, Vancouver, Canada) was stored at −80 °C in 15% glycerol stocks. Before the assay, the bacteria were grown on LB agar plates (Oxoid, Hampshire, UK) cultured overnight at 37 °C in an incubator IB013 (JeioTech, Lab Companion, Daejeon, Korea). The plates were examined for bacteria chemiluminescence on the UVITEC Alliance Q9 biomolecular imaging apparatus (Figure S5, Uvitec, Cambridge, UK). LB medium (30 mL) in sterile Falcon tube was inoculated with PA-Lux from the agar plate and incubated at 37 °C for 16 h at 200 RPM in MIULAB shaking incubator (Hangzhou Miu Instruments Co., Ltd., Hangzhou, Zhejiang, China). Then, 1 mL of the culture was transferred to 10 mL of fresh LB medium supplemented with 20 mM glucose (Sigma-Aldrich, Darmstadt, Germany) and cultured under the same conditions for 2 h. The optical density of the resulting bacterial suspension at 600 nm is usually 0.3–0.4 (1 cm path length cuvette, visible spectrometer METASH V-5000, Shanghai Metash Instruments Co., Ltd., Songjiang District, Shanghai, China). This PA-Lux suspension was used to determine the antimicrobial activity of plant extracts.

### 4.6. Determination of Antimicrobial Activity

Two-fold serial dilutions of plant extracts in LB medium were performed in 96-well sterile black microtiter plates (COSTAR 96 flat bottom black polystyrene plates, Corning, Tewksbury, Massachusetts, USA), applying 100 μL of bioluminescent PA-Lux (2 × 10^7^ CFU) to each well containing 100 μL of diluted extract in LB medium (see Section 4.5.). During incubation at 37 °C in a TECAN Infinite 200 PRO microplate luminescence reader, bacterial luminescence was determined using five 20 min kinetic cycles (Tecan Group Ltd., Männedorf, Switzerland). The luminescence determination was always preceded by orbital shaking (amplitude 1 mm) for 10 s. The reader was set up for the integration time of 1500 ms and settle time of 10 ms using Tecan i-control software (version 1.8.50.0). All tests were performed in triplicates. LB medium served as a negative control (made in quadruplicate) and diluted extracts were measured as blank. Serial dilutions of sodium azide (0.06–15 mM) were used as a positive control of antimicrobial effect. MIC50 was determined as a concentration of lyophilizate or evaporate of plant material (mg/mL) in incubation mixture with PA-Lux that led to luminescence (viability) decrease to 50%.

### 4.7. Statistics

All experiments were repeated in two biologically independent replicates, performing all measurements in at least triplicates. Data were analyzed by One Way ANOVA (Holm-Sidak method) and t-test; differences were considered significant at *p* ≤ 0.05 in SigmaPlot 12.0 and GraphPad Prism 8.0.

## 5. Conclusions

All tested parts of the medicinal plants *O. vulgare* and *A. eupatoria* contained a variety of secondary metabolites and showed antioxidant properties and the ability to inhibit the growth of *P. aeruginosa*. Although the shoots of both plants are traditionally used, the highest antibacterial activity was surprisingly found in the ethanolic extract of the roots and flowers of *O. vulgare*. Extracts of *A. eupatoria* had a weaker effect on bacteria, but ethanol extract of leaves, flowers and also roots reduced PA-Lux bioluminescence (viability) up to 50%. The analysis of secondary metabolites showed that the phenolic acids chlorogenic, sinapic, *p*-coumaric, quinic, protocatechuic, and the flavonoids rutin, quercetin, chrysin are most likely involved in the antibacterial activity of *O. vulgare* extracts, in synergy with other substances. Thus, our results show that not only essential oils but also aqueous and ethanol extracts of medicinal plants, containing a wide range of phenolic compounds, exert significant antibacterial effects.

## Figures and Tables

**Table 1:** Minimal inhibitory concentration (MIC50) values of water or 60% ethanolic extracts of *A. eupatoria* and *O. vulgare*.

Plant/Extraction Method	Plant Part	MIC50 (Lyophilizate) [mg/mL]	MIC50 (Evaporate) [mg/mL]
*A. eupatoria* water extract	flowers	**10**	**17.7 ± 2.6**
	leaves	15	16.7 ± 0.0
	stems	21	10.1 ± 3.6
	roots	20	16.5 ± 0.0
*A. eupatoria* 60% ethanolic extract	flowers	**52**	**24.1 ± 2.7**
	leaves	**55**	**19.7 ± 3.0**
	stems	83	14.3 ± 0.4
	roots	**55**	**17.3 ± 0.0**
*O. vulgare* water extract	flowers	18	10.2 ± 2.7
	leaves	15	18.2 ± 0.0
	stems	21	7.1 ± 1.7
	roots	**8**	**12.4 ± 0.1**
*O. vulgare* 60% ethanolic extract	flowers	**7**	**4.5 ± 0.0**
	leaves	**29**	**14.6 ± 1.2**
	stems	87	28.5 ± 0.7
	roots	**4**	**2.3 ± 0.4**

MIC50 was expressed as a concentration of lyophilizate or evaporate of plant material (mg/mL) in incubation mixture with PA-Lux that led to luminescence (viability) decrease to 50%. Plant extracts that reached inhibition ≤ 50% of PA-Lux viability experimentally are highlighted in bold; for other extracts, MIC50 values were derived from their highest measured inhibition. Abbreviations: PA-Lux, luminescent strain of *P. aeruginosa*.

**Table 2:** Analysis of secondary metabolites in *A. eupatoria* and *O. vulgare* extracts prepared in water or 60% ethanol. The quantity is expressed as nmol per gram of dry plant matter.

			nmol/g (Dry Weight)																						
Plant	Part	Extraction	CAF	CHLG	CIN	CRY	DMY	FER	GAL	HES	MOR	NAR	PCA	PHE	PRO	QA	QRC	QUE	RES	RUT	SAL	SHI	SIN	SYR	VAL
*A. eupatoria*	leaves	water	419 ± 0	10 ± 0	<LOD	<LOD	24 ± 4	<LOD	<LOD	<LOD	<LOD	<LOD	111 ± 4	179 ± 17	49480 ± 0	2530 ± 0	<LOD	<LOD	<LOD	<LOD	<LOD	161 ± 0	<LOD	<LOD	<LOD
	flowers		708 ± 0	28 ± 0	<LOD	<LOD	6 ± 1	<LOD	<LOD	<LOD	<LOD	1 ± 0	89 ± 3	148 ± 8	24070 ± 0	1903 ± 173	<LOD	<LOD	<LOD	<LOD	<LOD	179 ± 3	<LOD	<LOD	<LOD
	stems		338 ± 0	3 ± 0	<LOD	<LOD	<LOD	<LOD	<LOD	<LOD	<LOD	2 ± 0	<LOD	37 ± 0	52 ± 1	2023 ± 107	<LOD	<LOD	<LOD	<LOD	<LOD	31 ± 1	<LOD	<LOD	14 ± 4
	roots		1870 ± 0	3 ± 0	<LOD	<LOD	<LOD	<LOD	20 ± 0	<LOD	2.2 ± 0	<LOD	8 ± 0	21 ± 0	106 ± 1	469 ± 56	<LOD	<LOD	<LOD	<LOD	<LOD	34 ± 8	12 ± 0	<LOD	27 ± 2
	leaves	60% EtOH	333 ± 5	<LOD	<LOD	<LOD	15 ± 1	<LOD	<LOD	<LOD	<LOD	<LOD	19 ± 0	27 ± 10	10730 ± 0	860 ± 61	<LOD	<LOD	<LOD	<LOD	<LOD	41 ± 2	<LOD	<LOD	3 ± 0
	flowers		399 ± 6	17 ± 0	<LOD	<LOD	13 ± 2	<LOD	<LOD	<LOD	<LOD	0.2 ± 0.1	100 ± 42	31 ± 10	<LOD	665 ± 55	<LOD	<LOD	<LOD	<LOD	<LOD	26 ± 1	<LOD	<LOD	1 ± 0
	stems		340 ± 0	3 ± 0	2 ± 0	<LOD	<LOD	<LOD	2 ± 0	<LOD	<LOD	1.5 ± 0.1	<LOD	10 ± 0	169 ± 68	637 ± 81	<LOD	<LOD	<LOD	<LOD	<LOD	28 ± 7	<LOD	33 ± 3	5 ± 0
	roots		935 ± 0	<LOD	1.2 ± 0	<LOD	0.2 ± 0	<LOD	5 ± 1	<LOD	<LOD	<LOD	10 ± 0	2 ± 0	220 ± 0	203 ± 12	<LOD	<LOD	<LOD	<LOD	<LOD	14 ± 1	3 ± 0	<LOD	9 ± 0
*O. vulgare*	leaves	water	<LOD	<LOD	<LOD	249 ± 0	<LOD	5 ± 0	289 ± 0	<LOD	<LOD	16 ± 0	40 ± 13	108 ± 16	26 ± 6	732 ± 139	6086 ± 268	<LOD	3 ± 2	<LOD	<LOD	<LOD	<LOD	41 ± 19	36 ± 0
	flowers		<LOD	5 ± 0	<LOD	1014 ± 59	1.3 ± 0	<LOD	15 ± 0	5 ± 0	1.4 ± 0	<LOD	27 ± 0	124 ± 1	30 ± 1	852 ± 19	5295 ± 522	2 ± 0	<LOD	5 ± 0	7 ± 0	<LOD	<LOD	<LOD	<LOD
	stems		<LOD	20 ± 0	<LOD	24 ± 9	3 ± 1	<LOD	2224 ± 0	144 ± 0	3.7 ± 0	<LOD	2 ± 1	30 ± 0	6 ± 0	133 ± 8	228 ± 93	12 ± 0	6 ± 0	138 ± 0	43 ± 0	61 ± 0	<LOD	<LOD	<LOD
	roots		<LOD	556 ± 0	<LOD	17 ± 3	9 ± 0	<LOD	439 ± 97	303 ± 0	5 ± 0	5 ± 0	5 ± 1	13 ± 0	112 ± 7	8 ± 0	183 ± 10	14 ± 0	2.5 ± 0	290 ± 0	12 ± 0	48 ± 0	13 ± 1	<LOD	<LOD
	leaves	60% EtOH	<LOD	<LOD	<LOD	298 ± 0	<LOD	2 ± 0	<LOD	<LOD	<LOD	<LOD	17 ± 7	76 ± 0	17 ± 0	149 ± 26	1352 ± 206	<LOD	<LOD	<LOD	<LOD	<LOD	<LOD	<LOD	<LOD
	flowers		<LOD	4 ± 0	<LOD	1254 ± 0	1 ± 0	<LOD	<LOD	10 ± 0	0.8 ± 0	<LOD	7 ± 1	68 ± 0	33 ± 0	197 ± 23	1215 ± 51	1 ± 0	<LOD	9 ± 0	5 ± 0	<LOD	<LOD	<LOD	3 ± 0
	stems		<LOD	7 ± 0	<LOD	54 ± 18	<LOD	2 ± 0	<LOD	31 ± 0	15 ± 0	<LOD	3 ± 1	30 ± 0	8 ± 2	45 ± 1	<LOD	25 ± 0	1.9	30 ± 0	<LOD	17 ± 10	<LOD	<LOD	16 ± 0
	roots		15 ± 0	208 ± 0	<LOD	26 ± 0	<LOD	0.4 ± 0	<LOD	53 ± 0	25 ± 0	2 ± 0	3 ± 1	4 ± 0	30 ± 1	4 ± 0	7 ± 0	40 ± 0	0.2 ± 0	50 ± 0	4 ± 0	22 ± 0	4 ± 0	<LOD	10 ± 3

Abbreviations: CAF, caffeic acid; CHLG, chlorogenic acid; CIN, *trans*-cinnamic acid; CRY, chrysin; DMY, dihydromyricetin; FER, trans-ferulic acid; GAL, gallic acid; HES, hesperidin; MOR, morin; NAR, naringin; PCA, *p*-coumaric acid; PHE, phenylalanine; PRO, protocatechuic acid; QA, quinic acid; QRC, quercitrin; QUE, quercetin; RES, resveratrol; RUT, rutin; SAL, salicylic acid; SHI, shikimic acid; SIN, sinapic acid; SYR, syringic acid; VAL, vanillin; <LOD, under the limit of detection.

## Data Availability

Raw research data can be shared on e-mail request.

## Supplementary Materials

The following supporting information can be downloaded at: https://www.mdpi.com/article/10.3390/molecules28031019/s1, Figure S1: Dry weight expressed as percentage of fresh weight of *A. eupatoria* (black columns) and *O. vulgare* (striped columns). Values shown represent the mean ± SD (n = 3 biological series). Different letters next to each bar denote significant differences (p ≤ 0.05) among the plant groups according to ANOVA (Holm-Sidak method). Figure S2: Effect of sodium azide on PA-Lux bioluminescence after 1 hour incubation at 37 °C. IC50 was determined as 0.9 +/- 0.3 mM. Data were plotted in QtiPlot (ver 0.9.8.9). Figure S3: Pearson correlation coefficients from the comparison of determination of bioluminescence, flavonoids, phenolic compounds, minimal inhibitory concentration of lyophilizate in incubation mixture with PA-Lux for 50% of bioluminescence (MIC), and antioxidant capacity determined by FRAP. In picture A, there are correlation coefficients for water extracts and in picture B for ethanolic extracts for all plant parts (flowers, leaves, stems, and roots) of *A. eupatoria* and *O. vulgare*. Figure S4: Correlation analysis among all identified secondary metabolites in water or 60% ethanol extracts of *A. eupatoria* and *O. vulgare*. Data were plotted using the Seaborn library for making statistical graphics in Python (Waskom, 2021). Abbreviations: CAF, caffeic acid; CHLG, chlorogenic acid; CIN, *trans*-cinnamic acid; CRY, chrysin; DMY, dihydromyricetin; FER, trans-ferulic acid; GAL, gallic acid; HES, hesperidin; MOR, morin; NAR, naringin; PCA, *p*-coumaric acid; PHE, phenylalanine; PRO, protocatechuic acid; QA, quinic acid; QRC, quercitrin; QUE, quercetin; RES, resveratrol; RUT, rutin; SAL, salicylic acid; SHI, shikimic acid; SIN, sinapic acid; SYR, syringic acid; VAL, vanillin; <LOD, under the limit of detection. Figure S5: Bioluminescence of PA-Lux. Petri dish with PA-Lux (A), photographed with bioluminescence detection (B). Microtiter plate with PA-Lux incubated with *O. vulgare* water extract dilution series (C), photographed with bioluminescence detection using UVITEC Alliance Q9 biomolecular imaging apparatus (Uvitec Cambridge, UK). Abbreviations: PA- Lux, luminescent strain of *P. aeruginosa*. Figure S6: Calibration curves for total phenolics (A), total flavonoids (B), and antioxidant capacity by FRAP method (C). As a calibration standard, phenol was used for total phenolics, quercetin for total flavonoids and ascorbic acid for determination of antioxidant capacity. Abbreviations: FRAP, ferric ion reduction antioxidant power assay.
