# Supplementary material for: Effect of Agrimonia eupatoria L. and Origanum vulgare L. Leaf, Flower, Stem, and Root Extracts on the Survival of Pseudomonas aeruginosa
Source: Molecules. 2023 Jan 19;28(3):1019. doi: 10.3390/molecules28031019 (PMC9921178; doi:10.3390/molecules28031019)
Supplement: Supplementary file 1 [file molecules-28-01019-s001.zip › molecules-2160151-Supplementary.pdf]

SUPPLEMENT

Effect of *Agrimonia eupatoria* L. and *Origanum vulgare* L. leaf, flower, stem, and root extracts on the survival of *Pseudomonas aeruginosa*

Kateřina Bělonožníková[1], Eliška Sladkovská[1], Daniel Kavan[1], Veronika Hýsková[1], Petr Hodek[1], Daniel Šmíd[1], Helena Ryšlavá[1*]

[1] Department of Biochemistry, Faculty of Science, Charles University, Hlavova 2030, 128 43 Praha 2, Czech Republic

[*] *Correspondence to: Department of Biochemistry, Faculty of Science, Charles University, Hlavova 2030, Prague 2, CZ 128 40, Czech Republic.*
E-mail address: helena.ryslava@natur.cuni.cz (H. Ryšlavá).

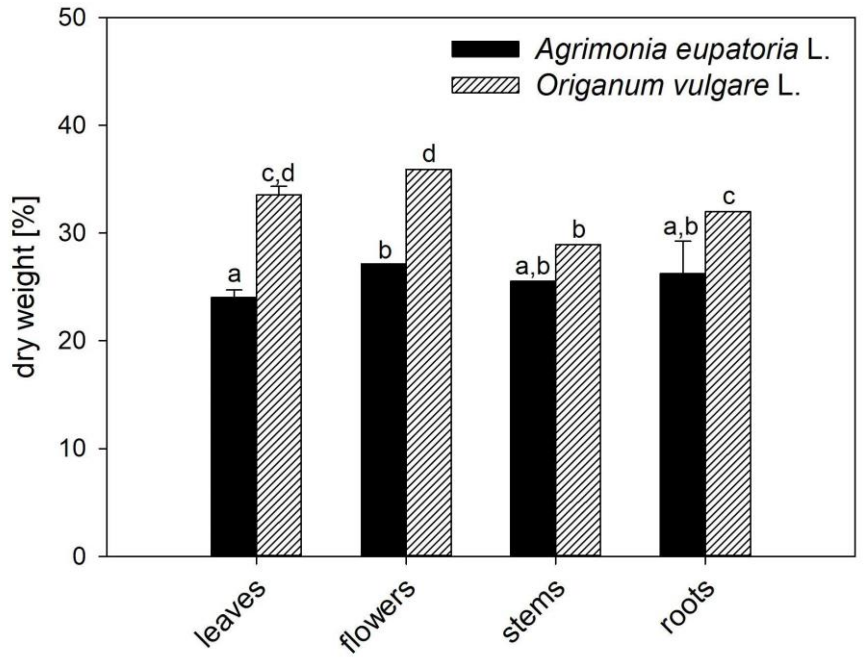

Figure S1: Dry weight expressed as percentage of fresh weight of *A. eupatoria* (black columns) and *O. vulgare* (striped columns). Values shown represent the mean±SD (n=3 biological series). Different letters next to each bar denote significant differences ($p \leq 0.05$) among the plant groups according to ANOVA (Holm-Sidak method).

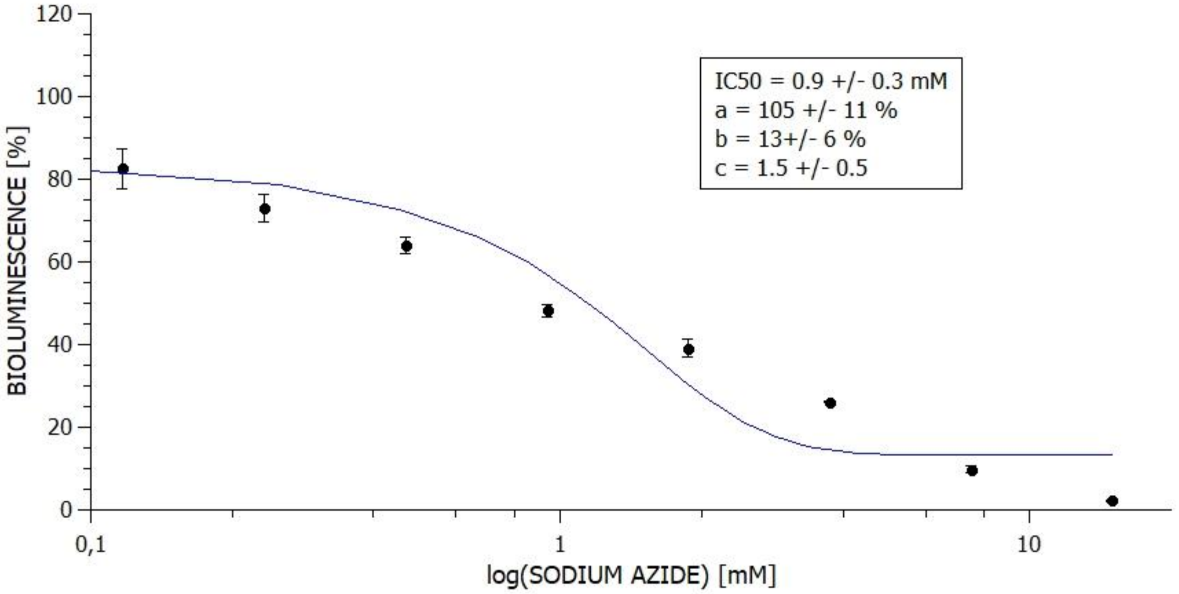

Figure S2: Effect of sodium azide on PA-Lux bioluminescence after 1 hour incubation at 37 ° C. IC50 was determined as 0.9 +/- 0.3 mM. Data were plotted in QtiPlot (ver 0.9.8.9).

A) Water extracts

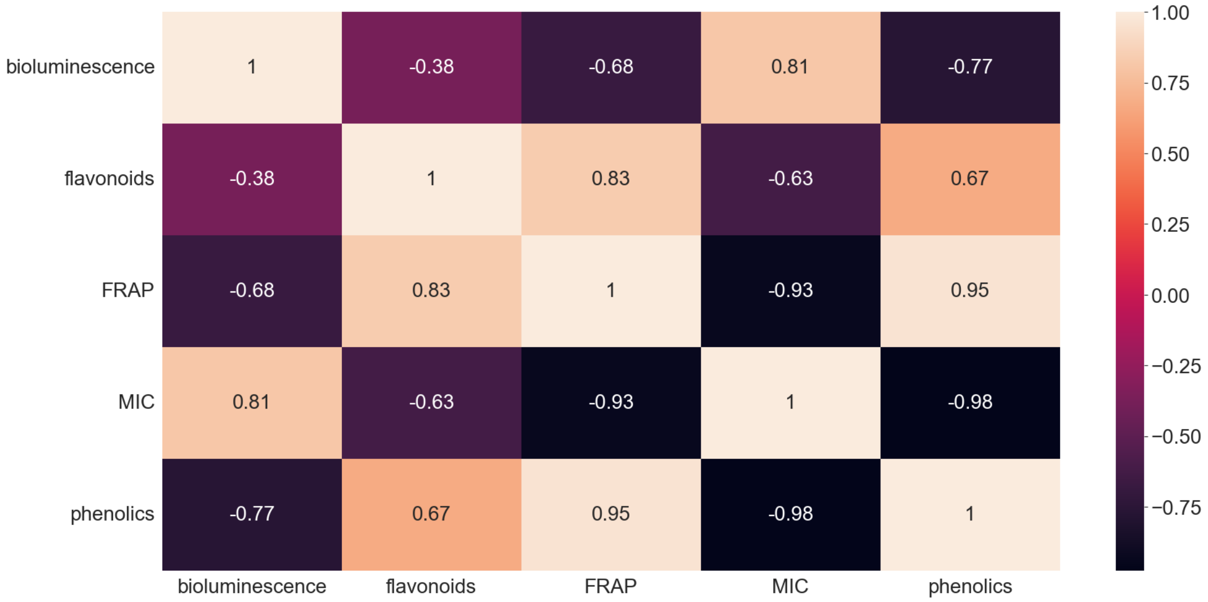

B) Ethanolic extracts

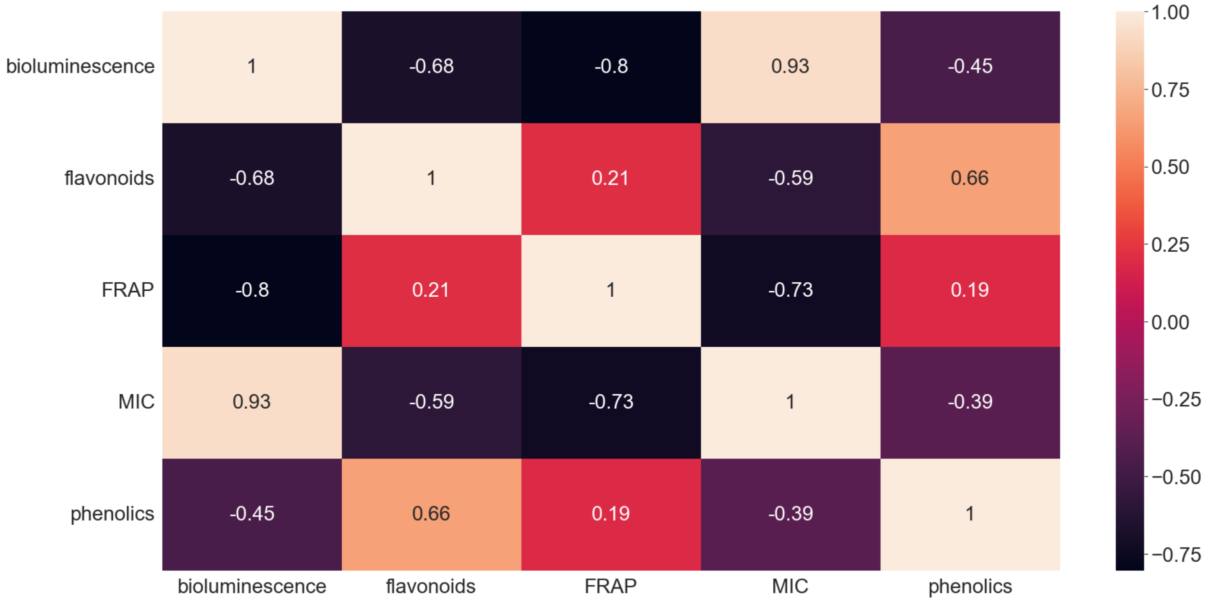

Figure S3: Pearson correlation coefficients from the comparison of determination of bioluminescence, flavonoids, phenolic compounds, minimal inhibitory concentration of lyophilizate in incubation mixture with PA-Lux for 50% of bioluminescence (MIC), and antioxidant capacity determined by FRAP. In picture A, there are correlation coefficients for water extracts and in picture B for ethanolic extracts for all plant parts (flowers, leaves, stems, and roots) of *A. eupatoria* and *O. vulgare*.

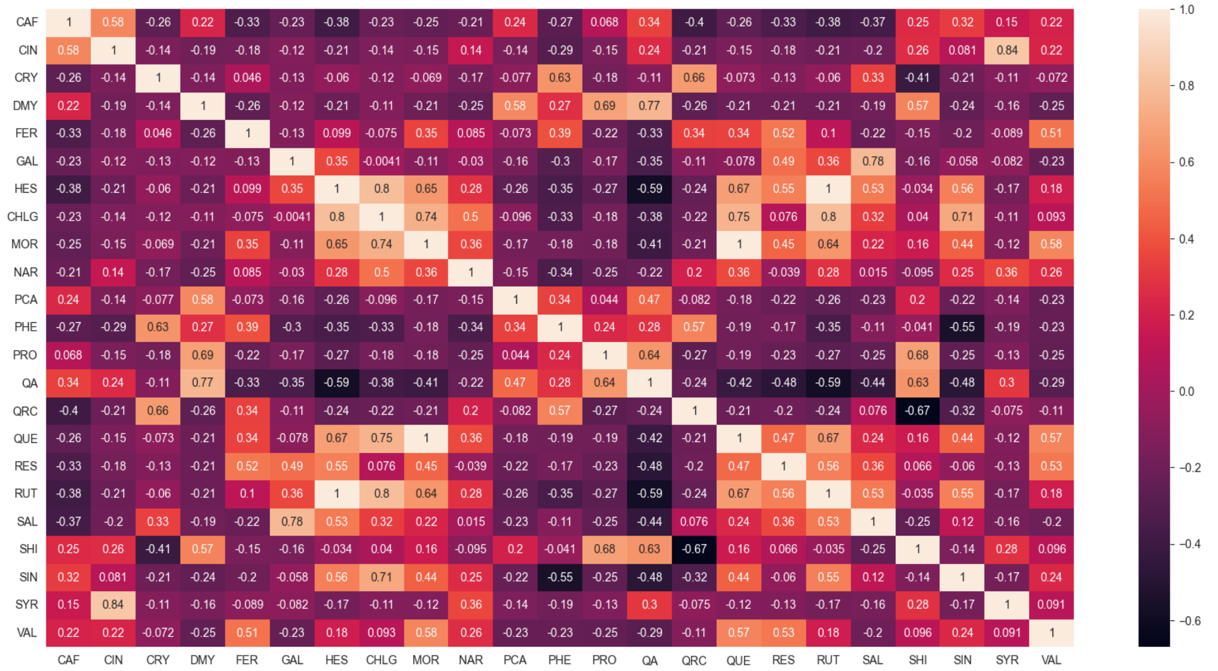

Figure S4: Correlation analysis among all identified secondary metabolites in water or 60% ethanol extracts of *A. eupatoria* and *O. vulgare*. Data were plotted using the Seaborn library for making statistical graphics in Python (Waskom, 2021). Abbreviations: CAF, caffeic acid; CHLG, chlorogenic acid; CIN, *trans*-cinnamic acid; CRY, chrysin; DMY, dihydromyricetin; FER, trans-ferulic acid; GAL, gallic acid; HES, hesperidin; MOR, morin; NAR, naringin; PCA, *p*-coumaric acid; PHE, phenylalanine; PRO, protocatechuic acid; QA, quinic acid; QRC, quercitrin; QUE, quercetin; RES, resveratrol; RUT, rutin; SAL, salicylic acid; SHI, shikimic acid; SIN, sinapic acid; SYR, syringic acid; VAL, vanillin; <LOD, under the limit of detection.

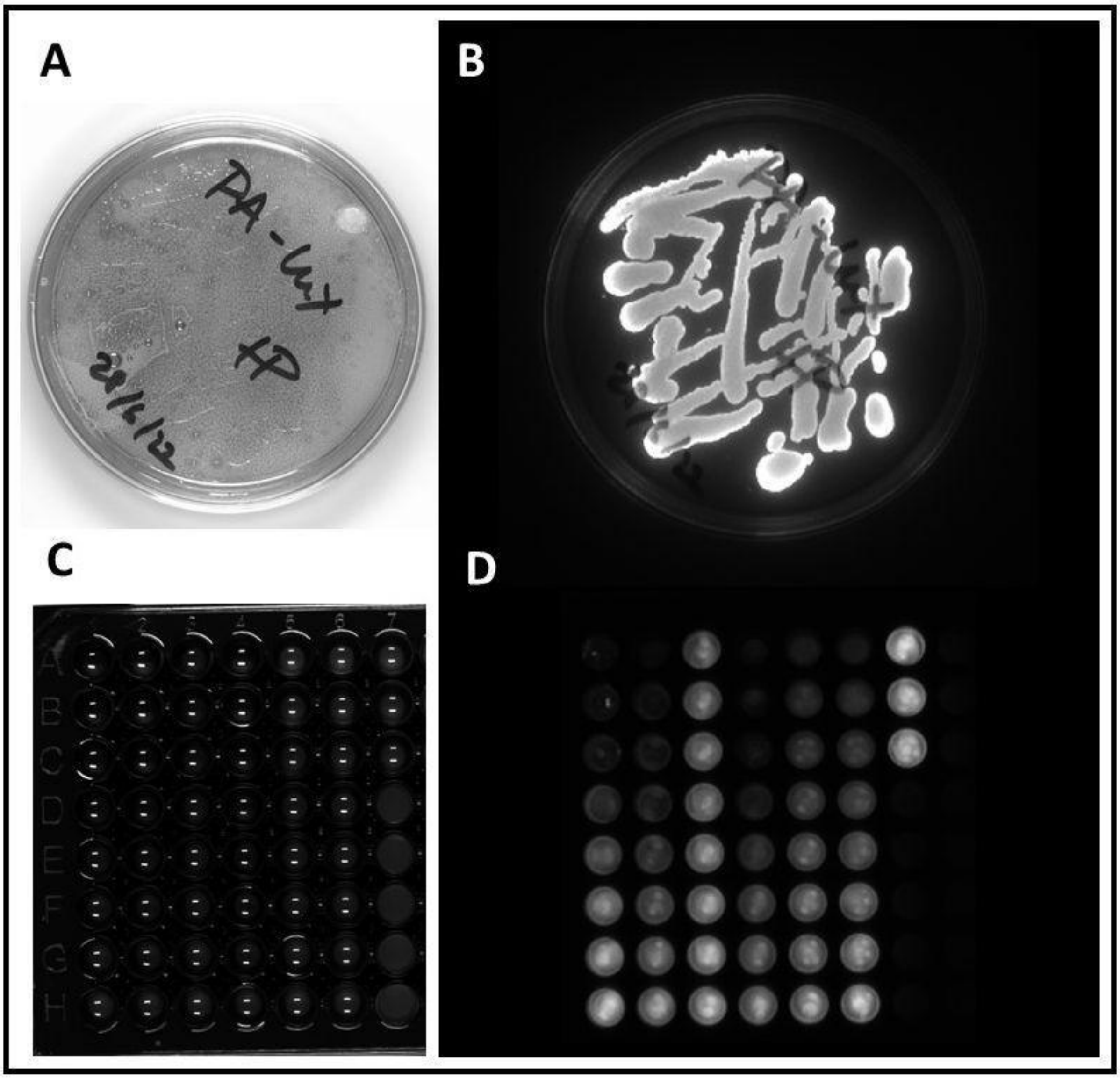

Figure S5: Bioluminescence of PA-Lux. Petri dish with PA-Lux (A), photographed with bioluminescence detection (B). Microtiter plate with PA-Lux incubated with *O. vulgare* water extract dilution series (C), photographed with bioluminescence detection (D) using UVITEC Alliance Q9 biomolecular imaging apparatus (Uvitec Cambridge, UK). Abbreviations: PA-Lux, luminescent strain of *P. aeruginosa.*

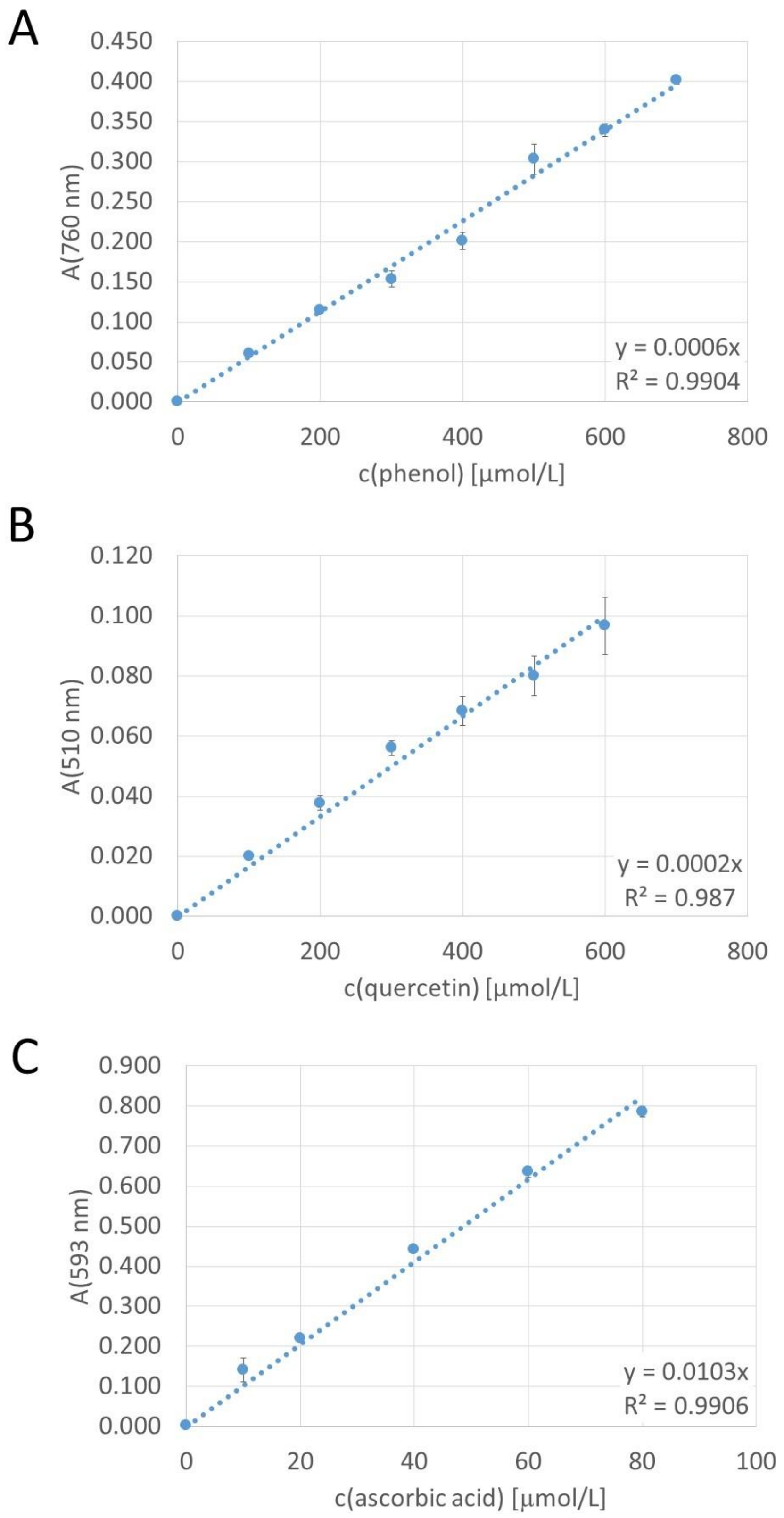

Figure S6: Calibration curves for total phenolics (A), total flavonoids (B), and antioxidant capacity by FRAP method (C). As a calibration standard, phenol was used for total phenolics, quercetin for total flavonoids and ascorbic acid for determination of antioxidant capacity. Abbreviations: FRAP, ferric ion reduction antioxidant power assay.
